# Attitudes of the Surgical Team Toward Artificial Intelligence in Neurosurgery: International 2-Stage Cross-Sectional Survey

**DOI:** 10.1016/j.wneu.2020.10.171

**Published:** 2021-02

**Authors:** Hugo Layard Horsfall, Paolo Palmisciano, Danyal Z. Khan, William Muirhead, Chan Hee Koh, Danail Stoyanov, Hani J. Marcus

**Affiliations:** 1 Department of Neurosurgery, National Hospital for Neurology and Neurosurgery, University College, London, United Kingdom; 2 Wellcome EPSRC Centre for Interventional and Surgical Sciences, University College, London, United Kingdom; 3 Department of Neurosurgery, Policlinico Gaspare Rodolico, Catania, Italy

**Keywords:** Artificial intelligence, Machine learning, Neurosurgery, Operative planning, Survey

## Abstract

**Background:**

Artificial intelligence (AI) has the potential to disrupt how we diagnose and treat patients. Previous work by our group has demonstrated that the majority of patients and their relatives feel comfortable with the application of AI to augment surgical care. The aim of this study was to similarly evaluate the attitudes of surgeons and the wider surgical team toward the role of AI in neurosurgery.

**Methods:**

In a 2-stage cross sectional survey, an initial open-question qualitative survey was created to determine the perspective of the surgical team on AI in neurosurgery including surgeons, anesthetists, nurses, and operating room practitioners. Thematic analysis was performed to develop a second-stage quantitative survey that was distributed via social media. We assessed the extent to which they agreed and were comfortable with real-world AI implementation using a 5-point Likert scale.

**Results:**

In the first-stage survey, 33 participants responded. Six main themes were identified: imaging interpretation and preoperative diagnosis, coordination of the surgical team, operative planning, real-time alert of hazards and complications, autonomous surgery, and postoperative management and follow-up. In the second stage, 100 participants responded. Responders somewhat agreed or strongly agreed about AI being used for imaging interpretation (62%), operative planning (82%), coordination of the surgical team (70%), real-time alert of hazards and complications (85%), and autonomous surgery (66%). The role of AI within postoperative management and follow-up was less agreeable (49%).

**Conclusions:**

This survey highlights that the majority of surgeons and the wider surgical team both agree and are comfortable with the application of AI within neurosurgery.

## Introduction

Artificial intelligence (AI) is the ability for a machine to think and learn. AI's potential disruption to workflows and boost in productivity stems from AI's ability to amass more experience than any single human over the course of their life. AI is also not subject to the preexisting human preferences. Combined with advances in computational power and data storage and the increasing availability of large high-quality digital data sets and machine learning frameworks, there has been an exponential increase in AI research, particularly in the health care sector.

The integration of AI into health care is likely to augment decision making, the ability to predict patient outcomes and also enhance efficiency.^1^^,^^2^ Several AI platforms have already been described within surgery, where they may improve decision making across all phases of care^3^ including preoperative diagnosis and surgical planning,^4^^,^^5^ intraoperative surgical workflow,^6^, ^7^, ^8^ providing postoperative reporting,^9^ and predicting postoperative outcome.^10^ Similar assistance has been reported in neurosurgery, especially within the subspecialties of oncology, spinal, and vascular surgery, by using platforms for image interpretation,^8^, ^9^, ^10^ preoperative and intraoperative planning,^11^, ^12^, ^13^, ^14^ and outcome prediction.^15^, ^16^, ^17^, ^18^, ^19^

In tandem with the practical development of AI platforms, rigorous evaluation of the proposed innovation must take place. The Idea, Development, Exploration, Assessment, Long-term study^20^ methodology provides a framework to evaluate and guide surgical innovation, through 5 distinct systematic stages. In addition to the evaluation of the technology, and before first-in-human studies, there must also be an assessment of the patients' and clinicians' perspectives on the acceptability of a device or technology.

Previously, our group published a 2-stage cross-sectional survey to better understand patients' and their relatives' attitudes toward AI and its role within neurosurgical procedures.^21^ The survey demonstrated the extent to which participants agreed with AI platforms designed to support the neurosurgeon, with the purpose of improving the surgical outcome and reducing the risks of complications. Responders in this survey largely disagreed with AI systems performing surgery entirely autonomously. Interestingly, respondents were comfortable with the concept of partially autonomous surgery, but less so when they were the patients undergoing partially autonomous surgery. In essence, respondents were comfortable with the use of AI systems to augment their care and support the surgeon.

The aim of this study was therefore to similarly evaluate the attitudes of surgeons and the wider surgical team toward the role of AI in neurosurgery.

## Methods

A cross-sectional, 2-stage, mixed-method (quantitative and qualitative) survey was performed. A qualitative survey was used to comprehensively appraise a surgical team's understanding of AI and its current utility in health care, in addition to examining their attitudes about AI applied in neurosurgery. A quantitative survey was then created to use themes identified from the initial qualitative survey to further explore attitudes of neurosurgeons using a case-based survey. Ethical approval was not required for this study as no patient or clinical data were collected, and the study was performed to plan and advise on future research.^22^ The surveys were administered as per recommended good survey practice,^23^ and results for both surveys were reported according to the American Association for Public Opinion Research standard definitions^24^: 1) questionnaires with 50%−80% of all applicable questions answered were considered partial responses; and 2) questionnaires with more than 80% of all applicable questions answered were considered complete responses.

### Qualitative Survey

The qualitative survey (Table 1) was created using Google Forms and distributed in June 2020. The survey was open for a 2-week period in June 2020. It was completed by all members of the neurosurgical team at an academic neurosciences unit including surgeons, anesthetists, nurses, and operating room practitioners. The survey was organized into 2 sections: 1) demographics; 2) four open-ended questions relating to AI.

**Table 1:** First-Stage Qualitative Survey: Open Questions

Q1	What do you know about artificial intelligence (AI) and its applications in everyday life?
Q2	What do you see as its roles in neurosurgery?
Q3	What do you think might be the advantages of AI in neurosurgery?
Q4	What do you think might be the disadvantages of AI in neurosurgery?

### Quantitative Survey

The quantitative survey (Table 2) was designed to further explore the major themes that emerged from the qualitative survey. The survey was distributed during a 2-week period in September 2020 to an international audience via social media (Twitter, Facebook, LinkedIn) and e-mail to members of neurosurgical societies. The survey was organized into 2 sections: 1) demographics and 2) six scenarios describing implementation of an AI system. The 6 scenarios were developed based on the thematic analysis of the initial qualitative survey and focused on the following themes: imaging interpretation and preoperative diagnosis, coordination of the surgical team, operative planning, real-time alert of hazards and complications, autonomous surgery, and postoperative management and follow-up. The responders used a 5-point Likert scale to answer 2 questions based on these scenarios: 1) Do you agree with this use of an AI system? (1 = strongly disagree; 2 = somewhat disagree; 3 = neither agree nor disagree; 4 = somewhat agree; 5 = strongly agree); and 2) How would you feel if you were involved in this case as part of the surgical team? (1 = extremely uncomfortable; 2 = somewhat uncomfortable; 3 = neither comfortable nor uncomfortable; 4 = somewhat comfortable; 5 = extremely comfortable).

**Table 2:** Second-Stage Quantitative Survey: Scenarios

Scenario 1	An AI system is designed to analyze and interpret radiologic images for identifying suspected lesions. A patient presents with persistent headaches and issues with balance. A plain CT demonstrates a well-circumscribed intracerebral lesion. An AI system interprets the scan and suggests a potential metastatic lesion. The AI system automatically books the patient a staging CT scan and an MRI to further delineate the lesion. It also suggests further management, such as steroids and transfer to a tertiary neurosurgical center automatically.
Scenario 2	An AI system is used to facilitate the optimum patient pathway for a patient requiring an operation. A patient is seen in the clinic and requires an urgent lumbar decompression and laminectomy. The AI system aggregates patient variables, comorbidities, age, and radiology findings and suggests a full patient pathway including operation date, preassessment, and any other personal requirements automatically. The AI system also takes into account other planning issues, such as full lists, and prioritizes the operating schedule.
Scenario 3	An AI virtual reality system is used in stereotactic neurosurgery to create 3D brain models and plan safe trajectories for electrode implantation. A patient suffering from recurrent drug-resistant incapacitating seizures might benefit from surgery. Stereoelectroencephalography is planned to identify the foci of seizure onset zone and whether this is amenable to surgery. A new AI system, adopting a virtual reality algorithm, is used to generate a 3D virtual anatomic model of critical structures and regions of interests from radiologic images. The AI system then uses the virtual reality−generated 3D model to plan safe trajectories and target regions for stereotactic electrode implanting. Further, during the insertion of electrodes, an intraoperative augmented reality interface appears to ensure safe placement of the electrodes.
Scenario 4	An AI system is used intraoperatively for real-time anatomic assessment and detection of potential risks. A patient undergoes surgery for removal of a suspected frontotemporal glioma adjacent to eloquent brain regions. The patient cannot tolerate an awake craniotomy. To minimize risk of surgical complications, a new AI system is adopted intraoperatively. During the operation, the system is connected to the camera of the surgical microscope and, using augmented reality, displays the principal anatomic structures and landmarks in real time. It further delineates the contour of the lesion and shows the safest surgical corridor. While approaching the lesion, the AI system notifies the surgeon of adjacent eloquent brain topography and signals an alert if there is high surgical risk to aid surgical intraoperative decision making.
Scenario 5	An AI system connected to an autonomous robotic arm has been developed to support the surgeon during complex spine surgery. An AI system has been developed to control a robotic arm to assist the surgeon in screw placement during spine surgery. The trajectories of the screws are automatically determined by using the AI system and preoperative spine scans. Further, the AI system is able to decide on the ideal screw length and material. The AI-guided robotic arm is operated to autonomously insert screws and rods as appropriate.
Scenario 6	An AI system is used to coordinate the follow-up of patients. A new AI-assisted follow-up system is used to monitor discharged patients who underwent neurosurgery. The AI system uses patients' baseline information and clinical data collected throughout their hospital admission. The AI system autonomously delivers telephone calls and interacts with patients via automated speech. The AI system ascertains evaluation of patients' satisfaction, recovery of surgical wound, postoperative complications, objective function, and patient-reported outcomes. The AI system then books outpatient follow-up appointments based on patient need and urgency.

AI, artificial intelligence; CT, computed tomography; MRI, magnetic resonance imaging; 3D, 3-dimensional.

### Data Analysis

The qualitative survey responses were analyzed to identify overarching themes. Thematic analysis methodology was guided by existing literature.^25^ Participants' knowledge about AI use in everyday life and its utility was assessed. For the subsequent 3 questions specifically relating to AI and neurosurgery (see Table 1), free text was analyzed from the answers and grouped together as codes. The codes were organized as themes. The perceived advantages and disadvantages of AI in neurosurgery were used to guide the development of 6 scenarios to further explore attitudes of the surgical team in the quantitative second-stage survey. The quantitative survey responses were numerically described using a 5-point Likert scale, and descriptive analysis performed. Demographic data on sex, age, profession, stage of training, country of residence, and previous experience of AI research were also analyzed descriptively.

## Results

### Qualitative Survey

In the first-stage survey (see Table 1), a total of 33 complete responses were collected. The responders identified as surgeons (14/33; 42%), anesthetists (10/33; 30%), nurses (3/33; 9%), operating room practitioners (4/33; 12%), and others (2/33; 6%). Most participants (19/33; 58%) acknowledged the role of AI in everyday life. Eleven more participants (11/33; 33%) found AI useful but stressed the importance of first understanding its limitations—primarily concerns about privacy and the potential negative impact of AI if implemented lacking oversight. Only 3 responders (3/33; 9%) were unaware of current AI applications in everyday life.

Thematic analysis identified numerous themes for AI's role within neurosurgery: 1) analysis of preoperative data (11/33; 33%); 2) preoperative assessment (11/33; 33%); 3) surgical augmentation, assistance, and automation (17/33; 52%); 4) coordination of the surgical team (6/33; 18%); 5) postoperative assessment and prognosis prediction (7/33; 21%); and 6) surgical workflow efficiency (4/33; 12%). Responders believed that AI could assist in diagnosis and data analysis (5/33; 15%), surgical planning (6/33; 18%), and surgical risk assessment (4/33; 12%). Further postulated roles for AI systems may include enhancing surgical technique and anatomic recognition (15/33; 63%) and early detection and assessment of intraoperative complications (4/33; 12%). AI was acknowledged to feasibly predict and improve outcomes (5/33; 15%). Regarding hospital admission and inpatient management, AI was considered to benefit patient care (6/33; 18%), support standardization of care by potentially reducing human error (5/33; 15%), and reduce, augment, and enhance workload (5/33; 15%). For example, AI enables prompt handover and information sharing for postoperative patient management, as well as organization of postdischarge follow-up visits. Lastly, responders highlighted the vital role of AI in education and research (7/33; 21%), such as virtual reality−based neuroanatomy teaching platforms for medical students and surgical trainees, AI-guided robotic surgical training, and radiogenomics algorithms for better understanding brain neurophysiology.

Our first-stage survey also explored responders' views on perceived disadvantages of AI within neurosurgery (see Table 1). In an operative environment, AI systems' complexity (5/33; 15%) and surgeons' reluctancy to change (2/33; 6%) were considered barriers to adoption. In relation to patient management, the responders were concerned about AI's nonspecific approach (7/33; 21%) and the potential loss of human touch (4/33; 12%), in addition to concerns about overreliance on AI systems (5/33; 15%). Further disadvantages related to the technical implementation of AI systems were the controversial reliability of software or data input (11/33; 33%), loss of surgical skill, additional “technical training” (11/33; 33%), responsibility and ethics (5/33; 15%), and cost of software or hardware (3/33; 9%).

### Quantitative Survey

We received 100 responses for the quantitative survey. The majority of responders were male (70/100; 70%) and aged between 31 and 40 years old (31/100; 31%), although 9% of responders were aged 61 years or older. We had responses from colleagues in 25 countries. The majority of responders were from the United Kingdom (70/100; 70%) and India (7/100; 7%). Surgeons were the most common responders (60/100; 60%), followed by anesthetists (18/100; 18%) and nurses (11/100; 11%). There were 5 responses from operating room practitioners and 6 responses from “other” including device representatives. Of the surgeons and anesthetists, the majority were consultants (surgeons: 62% consultants; anesthetists: 50% consultants). The most common subspecialties of the consultant neurosurgeons were oncology (16/37; 43%), pituitary and skull base (16/37; 43%), and spine (11/37; 30%). Of the 100 responders, 17% had been involved with previous AI research and 47% had never been involved with AI research or implementation of AI systems before. However, 36% of responders had no prior AI research experience but expressed interest in using AI in their own practice.

**Figure 1:**
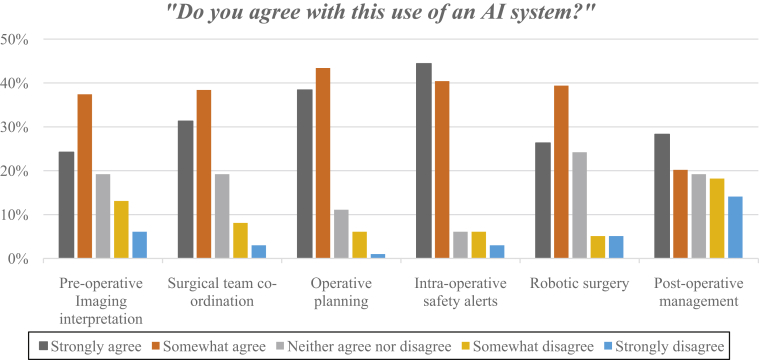
Responses from participants when asked “to what extent they agreed with the implementation of AI” in the given scenario, during our quantitative second-stage survey.

**Figure 2:**
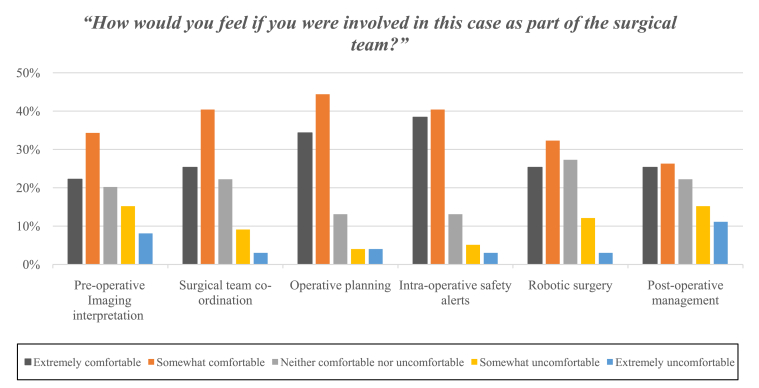
Responses from participants when asked “to what extent they felt comfortable as part of the surgical team” in the given scenario, during our quantitative second-stage survey.

Overall, the responders largely agreed and felt comfortable delivering patient care as part of the surgical team for the described implementation of AI (Figures 1 and 2). In the first scenario (see Table 2), the surgical team strongly or somewhat agreed with using AI for imaging interpretation and preoperative diagnosis (62%). Survey responders agreed with the application of artifical intellgence to assit with coordiantion of the surgical team (70%) (Scenario 2). AI's utility relating to operative planning (Scenario 3) and real-time alert of hazards and complications (Scenario 4) were the most agreeable scenarios to the responders. For AI used within operative planning, 81% strongly or somewhat agreed with the application. Similarly, for AI used to enhance real-time alert of hazards or complications, 85% strongly or somewhat agreed and 79% felt extremely or somewhat comfortable as part of the surgical team (see Figure 2). For AI used in the context of autonomous surgery (Scenario 5), 66% strongly or somewhat agreed with its use and 58% felt extremely or somewhat comfortable as part of the surgical team (see Figure 2). Concerning AI and its role within postoperative patient management and follow-up (Scenario 6), 49% strongly or somewhat agreed and 52% felt extremely or somewhat comfortable as part of the surgical team.

## Discussion

### Principal Findings

We present a comprehensive assessment of the attitudes of the surgeon and surgical teams toward the implementation of AI using an international 2-stage cross-sectional survey (see Tables 1 and 2). In the first-stage survey, we found that the majority of responders (91%) were aware of AI and its current everyday applications. Further, we elicited the apparent value of using AI within neurosurgery, such as improved surgery (63%) and enhanced diagnosis (33%). The first-stage survey also elicited rational concerns about AI and neurosurgery, such as the need to retrain, potential loss of surgical skill (33%), hesitancy about the reliability of software or hardware (33%), and proposed loss of human health care delivery (21%). Our first-stage survey also highlighted the importance of ethical considerations and AI (15%). In our second-stage survey assessing the attitudes of the neurosurgical team toward AI, we received 100 responses from 25 countries encompassing the entire neurosurgical team (neurosurgeons, anesthetists, nurses, and operating room practitioners). The 6 scenarios we developed assessed how strongly the survey participants agreed with the specific real-world application of AI and their comfort in being part of the team delivering patient care. Responders strongly or somewhat agreed with AI used for imaging interpretation (62%), operative planning (82%), coordination of the surgical team (70%), real-time alert of hazards and complications (85%), and autonomous surgery (66%) (see Figure 1). However, although the majority of respondents welcomed AI in a range of contexts, the role of AI in postoperative patient management was less favorable, with 49% strongly or somewhat agreeing to this use of AI. To the best of our knowledge, this is the first study exploring the views of international colleagues, encompassing the entire neurosurgical team, and real-world application of AI in neurosurgery.

### Comparison with Other Studies

Our study demonstrates that neurosurgical health care professionals believe implementation of AI could improve surgical workflows and support patient care. This perceived potential benefit is important, as positive general attitudes toward AI are postulated to feature in the overall acceptance of AI.^26^ However, valid concerns from the qualitative survey about “the loss of human touch” and postoperative patient management remains a pertinent point in relation to AI. This was also highlighted in our previous work assessing patient attitudes toward AI in neurosurgery, where most patients still preferred a human surgeon over an autonomous system.^21^ Thus our research supports existing literature^27^ that urges researchers to iteratively question “how do long-standing principles of medical ethics apply in this new world of technologic innovation?” The application of AI to health care is clearly a positive real-world utility of innovative technology, but potential harms of “algorithmic injustice” (e.g., predictive policing^28^ and facial recognition^29^) must be at the forefront of future AI research, regardless of discipline.^30^

In a recent Swiss study exploring the attitudes of neurosurgeons toward machine learning, Staartjes et al^31^ found that of the 362 participants surveyed, 29% were already implementing machine learning into their practice and a further 31% for research purposes. The most important reasons for applying machine learning to clinical practice were improved preoperative surgical decision making, objectivity in diagnosis, and improved anticipation of complications. These findings support our first-stage qualitative survey thematic analysis and further highlight the importance of ongoing research assessing feasibility and safety.

There is limited literature exploring the perception of AI elsewhere within health care. Sit et al explored the attitudes of U.K. medical students toward AI,^32^ with 89% of 484 survey responders agreeing with the important role AI will play in health care and 78% believing that AI learning should appear within their curriculum. Pinto Dos Santos et al^33^ similarly surveyed 263 German medical students and found that 77% felt AI would revolutionize radiology and 86% would improve radiology, in addition to 71% stating that AI should be included in medical training. In a Korean study, Oh et al^34^ surveyed 669 medical students and physicians and found that 83.4% had a favorable attitude toward AI and medicine. In a study of dermatologists, Polesie et al^35^ found that 78% of 1271 dermatologists surveyed agreed or strongly agreed that AI will improve their specialty, representing an overall optimistic attitude toward AI. Taken together, these studies are largely consistent with our findings, with responders acknowledging the potential utility AI offers across numerous specialties and the potential to improve patient care.

Our findings echo research into public attitudes toward AI: firstly, the general public “fears” AI replacing humans^36^ and, secondly, the concern about losing intelligent behavior in humans.^37^ We must continue to explore the foundation of these concerns and include multidisciplinary stakeholders including patients in the development of new surgical technologies. One such example by our own group is the iRobotSurgeon survey,^38^ which aims to explore public opinion about the responsibility and associated liability when surgical robotic systems cause harm. A study by Bossi et al^39^ has gone further, investigating the underlying reason for differences in human attitudes toward robotics. Here, they examined whether individual attitudes toward robots can be differentiated on the basis of default neural activity pattern during resting state, measured with electroencephalogram. Participants observed scenarios in which a humanoid robot was depicted performing various actions embedded in daily contexts. They found evidence that individual biases toward treating robots as either intentional agents or mechanistic artefacts can be detected at the neural level. Taken together, it is apparent that research groups are working to decipher how we can better understand our relationship with novel technologies—demonstrated through surveys^21^^,^^38^—and objective neural measures to understand how humans might explain the robot's “reasons” for actions.^39^

### Limitations

The present study has several limitations. The first-stage qualitative survey sample was performed in a single academic neurosurgical unit in a high-income setting, perhaps limiting the generalizability of the findings, and in addition adding a degree of bias to the scenario development. Similarly, despite our best efforts to perform an objective analysis, thematic analysis always contains an inherent degree of subjectivity.

The second-stage quantitative survey was primarily distributed on social media (Twitter, Facebook, LinkedIn), and we are therefore unable to record accurately the number of times the survey was distributed or report an accurate response rate. In addition, people using these platforms were more likely to be technology literate, introducing a selection bias. Additionally, our sample size of 100 responders for the second-stage survey is moderate.

The fact that the key study findings for the 2 stages are broadly consistent with each other, and with related literature, suggests they are likely to hold true.

## Conclusion

This 2-stage international survey represents an important further step in developing a rigorous evidence base to support the use of AI and neurosurgery. Taken together with our previous work, we have found that both patients and surgeons are receptive to the use of AI in neurosurgery. Furthermore, many members of the surgical team expressed motivation to participate in future AI adoption and research. To this end, frameworks of surgical device and technology innovation, such as The Idea, Development, Exploration, Assessment, Long-term study^20^ and CONSORT-AI,^40^ must be used to ensure transparent and robust translation of preclinical innovation into clinical practice. This will facilitate alleviating concerns of patients and the surgical team but also ensure we, as a community, are adhering to the ethical principles of modern medicine.
